# Within-Subject Variability of Interferon-g Assay Results for Tuberculosis and Boosting Effect of Tuberculin Skin Testing: A Systematic Review

**DOI:** 10.1371/journal.pone.0008517

**Published:** 2009-12-30

**Authors:** Richard N. van Zyl-Smit, Alice Zwerling, Keertan Dheda, Madhukar Pai

**Affiliations:** 1 Lung Infection and Immunity Unit, Division of Pulmonology and UCT Lung Institute, Department of Medicine, University of Cape Town, Cape Town, South Africa; 2 Department of Epidemiology & Biostatistics, McGill University, Montreal, Canada; 3 Montreal Chest Institute, Montreal, Canada; 4 Institute of Infectious Disease and Molecular Medicine, University of Cape Town, South Africa; 5 Centre for Infectious Diseases and International Health, University College Medical School, London, United Kingdom; National Institute for Infectious Diseases (INMI) L. Spallanzani, Italy

## Abstract

**Background:**

Variability in interferon-gamma release assays (IGRAs) results for tuberculosis has implications for interpretation of results close to the cut-point, and for defining thresholds for test conversion and reversion. However, little is known about the within-subject variability (reproducibility) of IGRAs. Several national guidelines recommend a two-step testing procedure (tuberculin skin test [TST] followed by IGRA) for the diagnosis of LTBI. However, the effect of a preceding TST on subsequent IGRA results has been reported in studies with apparently conflicting results.

**Methodology/Findings:**

We conducted a systematic review to synthesize evidence on within-subject variability of IGRA results and the potential boosting effect of TST. We searched several databases and reviewed citations of previous reviews on IGRAs. We included studies using commercial IGRAs, in addition to non-commercial versions of the ELISPOT assay. Four studies, fulfilling our predefined criteria, examined within-subject variability and 13 studies evaluated TST effects on subsequent IGRA responses. Meta-analysis was not considered appropriate because of heterogeneity in study methods, assays, and populations. Although based on limited data, within-subject variability was present in all studies but the magnitude varied (16-80%) across studies. A TST induced “boosting” of IGRA responses was demonstrated in several studies and although more pronounced in IGRA-positive (i.e. sensitized) individuals, also occurred in a smaller but not insignificant proportion of IGRA-negative subjects. The TST appeared to affect IGRA responses only after 3 days and may apparently persist for several months, but evidence for this is weak.

**Conclusions/Significance:**

Although reproducibility data are scarce, significant within person IGRA variability has been reported. If confirmed in more studies, this has implications for the interpretation of results close to the cut-point and for definition of conversions and reversions. Although the effect of TST on IGRA results is likely to be inconsequential in IGRA-positive subjects, in IGRA-negative subjects, the interpretation of results may be confounded by a preceding TST if administered more than 3 days prior to an IGRA.

## Introduction

In many countries with low incidence of tuberculosis (TB), serial (repeated) testing for latent TB infection (LTBI) is done for individuals at high risk of TB exposure. This is done, for example, in programs for screening of healthcare workers for LTBI as a component of TB infection control. Serial testing is also performed as part of TB contact investigations. Although widely used, the conventional tuberculin skin test (TST) has limitations in accuracy and reliability[1]. Furthermore, interpretation of serial TST results is particularly complicated because of non-specific variations in test results, boosting, conversions, and reversions [2], [3], [4].

Recently, the development of more specific, *in-vitro* assays for LTBI – interferon-gamma (IFN-γ release assays (IGRAs), has offered an alternative approach to LTBI diagnosis. IGRAs are blood tests that are based on IFN-γ release after stimulation by antigens (such as early secreted antigenic target 6 [ESAT-6], culture filtrate protein 10 [CFP-10] and TB7.7) that are more specific to *M. tuberculosis* than the purified protein derivative (PPD) used in TST. These assays are highly specific, especially in BCG vaccinated populations [5], [6]. IGRAs have features that make them ideal for serial testing: they are more specific than TST, they are *ex-vivo* assays and can be repeated any number of times without sensitization and boosting, the testing protocol does not require a second visit for reading, and unlike the TST, there is no need for a baseline two-step testing protocol. In all cases of a positive test, however, the patient will need to return for subsequent work-up and preventive therapy.

While some guidelines have recommended the use of IGRAs for serial testing[7], others have been more cautious [8], [9]. Some guidelines have suggested that TST may be replaced by IGRAs [7], while others have suggested initial testing with TST, with IGRA as a follow-up option to confirm TST results [8], [9]. Regardless of the approach, widespread use of IGRAs in serial testing is hampered by lack of evidence on several key questions (as reviewed elsewhere [10], [11]): a) What is the within-person reproducibility of T cell responses over time (in other words, what amount of variation is expected when IGRAs are repeated)? b) Given a certain degree of “inherent variability”, how does one interpret a single test result close to the assay cut point?; c) Will a TST boost or affect the results of subsequent IGRA testing and what is the optimum time gap between the two tests? d) What is an IGRA “reversion” and what threshold should be used to define reversion? e) What is the clinical significance and prognosis of an IGRA reversion? f) What is an IGRA “conversion” and what threshold (cut-off) should be used to define conversion? g) What is the prognosis (i.e. predictive value) of an IGRA conversion and will treatment of individuals with IGRA conversions reduce their risk of progression to active disease?

Unfortunately, data are lacking on these important questions and without such evidence, the results of serial IGRA testing will be difficult to interpret, especially if it is introduced in a routine testing program. In the past few years, there have been several attempts to answer at least two of the above questions: 1) reproducibility of IGRAs when repeated over time and 2) effect of TST on subsequent IGRA results. We performed a systematic review of these studies to inform policies and practices relevant to serial IGRA testing.

## Methods

### Objectives of the Review

Our systematic review aimed to synthesize evidence on two related questions: 1) What is the within-person reproducibility (i.e. variability) of T cell responses over time? 2) What is the effect of a tuberculin skin test on subsequent IGRA results and how do factors such as time interval after TST and baseline IGRA status affect the boosting results?

### Study Sources and Eligibility

We have previously published systematic and narrative reviews on IGRA accuracy and performance in various subgroups [5], [6], [12], [13], [14]). We updated the database searches that were done in previous systematic reviews and searched the literature for relevant IGRA studies (up to November 2009) that reported data on within-subject variability of IGRAs and/or data on effect of TST on subsequent IGRA results. We searched PubMed, Embase and Biosis and Web of Science, and reviewed citations of all original articles published in all languages.

The search terms used in database searching included: ((interferon-gamma release assay*) OR (T-cell-based assay*) OR (antigen-specific T cell*) OR (T cell response*) OR (T-cell response*) OR (interferon*) OR (interferon-gamma) OR (gamma-interferon) OR (IFN) OR (elispot) OR (ESAT-6) OR (CFP-10) OR (culture filtrate protein) OR (Enzyme Linked Immunosorbent Spot) OR (Quantiferon* OR Quantiferon-TB Gold)) AND ((tuberculosis OR mycobacterium tuberculosis)).

In addition to database searches, we reviewed bibliographies of previous reviews and guidelines on IGRAs, and also screened the citations of relevant original articles. Experts in the field and commercial test manufacturers were also contacted to obtain relevant citations. No language restrictions were imposed and full-length papers as well as conference abstracts were included (to limit potential publication bias).

We included studies of QuantiFERON-TB Gold (QFT-G, also known as QFT-2G), QuantiFERON-TB Gold In-Tube (QFT-GIT, also known as QFT-3G) [Cellestis Limited, Victoria, Australia], and the T-SPOT.TB [Oxford Immunotec, Oxford, UK] or its pre-commercial ELISPOT version. Where relevant, we included in-house, short-incubation (overnight) IFN-γ assays with RD1 antigens as well, to increase the number of relevant studies.

For studies assessing reproducibility (defined as within-subject repeatability over time, under similar conditions), the study had to have repeated (at least two) IGRA assays (same IGRA) done on the same group of subjects, preferably in a setting with limited TB exposure and without an antecedent TST within 6 months. If reproducibility was done in a high TB incidence setting where exposure-related changes are likely, then repeat tests should have been done over a short period of <6 weeks (to avoid the confusion between conversions (or new infections) and natural variations in T-cell responses). For studies assessing boosting of IGRA results due to a prior TST, the study sample must have had at least one IGRA assay done before and after tuberculin skin testing and not performed in the context of a contact or outbreak study in a high incidence setting (again, to avoid the confusion between true conversion and boosting).

We did not consider reproducibility data where two or more tests were done on the same sample at the same time (e.g. two tests done using samples from the same blood draw); this would not have been informative for our objective of determining the within-person variability when the test is repeated over time (serial testing). Also, we did not consider other forms of reproducibility data, such as inter-laboratory variation, variations between lab technologists, batch-to-batch variations, variations due to different incubation times, etc.

### Study Selection and Data Extraction

Two independent reviewers (RVZS & AZ) perused searches and selected articles meeting our inclusion criteria. One reviewer (RVZS) abstracted data, using a standardized template, regarding patient characteristics and test characteristics and outcomes, and these data were independently verified by a second reviewer (AZ). Where necessary, study authors were contacted for additional or missing information.

### Data Synthesis and Analysis

For each study, we extracted data on reproducibility and summarized the results in tables. Data on boosting were separately extracted and tabulated. Because of heterogeneity in study designs, time intervals between tests, study populations and assays, we decided to not perform pooled analyses (meta-analyses).

## Results

### Characteristics of Included Studies

**Figure 1:**
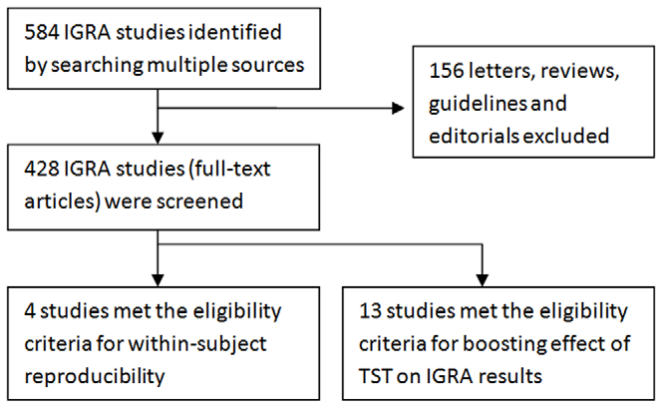
Study selection flow chart.

Our literature searches identified a total of 428 studies on IGRAs (commercial and in-house), excluding reviews, editorials, letters (not containing original data), and guidelines. After reviewing these, we identified 4 studies [2], [3], [15], [16] on within-person variability, and 13 studies [2], [17], [18], [19], [20], [21], [22], [23], [24], [25], [26], [27], [28] on potential boosting of IGRA results by TST (Figure 1 shows the study selection flow chart). In all, these studies included a total of 1460 subjects. The average number of subjects per variability study was 46 (range 14 to 117). The average number of subjects per boosting study was 91 (range 9 to 530). Of the total of 13 studies, 2 (14%) were done in high TB incidence settings, and 86% in low incidence settings (although several of these studies included immigrants from high burden countries). The populations included in these studies were heterogeneous, although several studies used healthcare workers as volunteers.

### Within-Person Variability Results

Table 1 shows the results of the reproducibility studies. As shown in the table, a total of four studies were included. [2], [3], [15], [16] Although some other studies reported the reproducibility of IGRA assays, these were not included, as a TST had been performed at the time of the initial IGRA [22], [29] and therefore reproducibility results could have been impacted by TST-induced changes in IGRA results. Three studies were performed in a high burden setting (India and South Africa) and one in a low burden setting (USA). Comparison of high vs. low burden settings was not possible as the American study is ongoing and only limited data were available for inclusion. Only one study directly compared the variability of the T-SPOT.TB and QFT-GIT in a head to head study. [2]

**Table 1:** Studies on within-person variability of Interferon gamma release assays in high and low burden countries.

Study, Reference, Year	Country (TB Prevalence)	Participants	TB Exposure during study	BCG status	IGRA	Time points (days)	Internal quality control	Study results summary (within-subject variability)	Comment
Veerapathran et al [3] 2008	India (high)	14 HCWs (clinical and laboratory workers)*	Likely but all tests were done within a 2-week period	All vaccinated	QFT-GIT	0, 3, 9, 12	Yes	Over a 2 weeks period, 2 of 14 persons had a QFT reversion.With quantitative results, an increase in 16% of IFN-γ response was considered within the ‘normal’ expected within subject variability.	Subjects who had conversions or reversions had initial values close to the cut point
Van Zyl-Smit et al [2] 2009	South Africa (High)	26 HCWs and low risk volunteers+	Likely but all tests were done within a 3 week period	All vaccinated	QFT-GITT-SPOT.TB	0, 7, 14, 21	Yes	Over a 3 week period, 7 of 26 persons had a conversion or reversion (1 QFT and 6 TSPOT TB).With quantitative results, a change of ±80% of any given IFN-γ response (QFT-GIT) or ±3 spots (T-SPOT.TB) was considered to fall within the ‘normal’ expected within subject variability.	Subjects who had conversions or reversions had initial values close to the cut point
Detjen et al [16] 2009	South Africa (High)	27 HCW's (clinical and laboratory workers)*	Likely but all tests were done within a 3-day period	all vaccinated	QFT-GIT	0, 3	Yes	Over a 3-day period, no changes in qualitative results were noted for 15 persons. With quantitative results, considerable intra-individual variability occurred in the magnitude of IFN-γ responses; intra-class correlation was 0.80.	
Belknap et al [15] 2009 (abstract)#	USA (low)	117 HCWs	Unlikely and all tests were done within a 3 week period	Unknown	QFT-GITT-SPOT.TB	0, 7-21	Yes	Over a 3 week period, 7 of 117 (6%) persons had a conversion or reversion with QFT-GIT and 8 of 105 (7.6%) with T-SPOT.TB	Quantitative results not yet available

* India and South Africa are high prevalence TB countries with high risk of exposure to health care workers (HCW). HCW's were divided into two groups – medical doctors or laboratory workers.

+South Africa is a high prevalence TB country with high risk of exposure to health care workers (HCW). HCW's were stratified into: High risk (daily potential TB exposure) Medium risk (HCW, but no daily expected TB exposure) Low risk group (pre-clinical medical students and non-clinical volunteers).

#updated preliminary data presented at the Second Global Symposium on IGRAs. Daley C. Evaluation of interferon-g release assays in the diagnosis of latent TB infection in US healthcare workers: preliminary results of Task Order #18. 31 May 2009; Second Global Symposium on IGRAs, Dubrovnik, Croatia2009.

It was evident from the four published studies that the statistical analysis of within-subject variability is complex as multiple samples are taken in multiple individuals at multiple time points. Although kappa statistics can be used to analyse concordance in dichotomous results, to interpret the variability in continuous variables more complex statistical modelling was used in the studies.

The study in India (4 repeat tests over a 2 week period) reported a variability of 16% in IFN-γ responses as measured by the QFT GIT to be within the bounds of statistical probability[3]. The other study to report variability in the continuous results performed in South Africa (4 tests over 3 weeks) reported a variability of 80% in IFN-γ responses (QFT GIT) and 3 spots T-SPOT.TB to be the 95% confidence interval for within-subject variability[2]. In both these studies, subjects who spontaneously converted or reverted had initial test results that were close the assay cut point. The study by Detjen et al. repeated the QFT GIT on day one and three and showed no changes in quantitative (dichotomous) results although there was considerable variability in the continuous IFN-g values (intra-class correlation of 0.80) [16].

Overall, although only 4 small reproducibility studies were identified, all showed variations in IFN-γ responses, even over short periods of time, and even in low exposure settings. The data suggest that spontaneous conversions and reversions can potentially occur during serial testing, even in the apparent absence of any exposure over a short time period. However, given the limited evidence, these observations require further confirmation in well-powered studies.

### Boosting Effect of TST on IGRA Results

Table 2 shows the results of the boosting studies. As shown in the table, a total of 13 studies have examined the impact of TST on subsequent IGRA results. Only one of these studies was performed in a high burden country although many of the studies in low burden countries recruited immigrants or HCWs who could be considered to have higher risk prior of TB exposure than the normal population.

**Table 2:** Studies on boosting effect of tuberculin skin test on IGRA results.

Study, Reference, Year	Country/population recruited (TB burden)	Participants	TST	IGRA	Time points (days after TST administration)	Study results summary	Comment	Study conclusion on boosting
van Zyl-Smit et al [2] 2009	South AfricaHCW's and healthy volunteers (High)	24	2TU RT23	QFT-GITTSPOT.TB	0,3,7,28,84	Day 3: no categorical changesDay 7: Significant increase in mean IFN-γ, QFT-GIT 1/12 (8%) negative to positive, 5/8 (62.5%) positive ↑ in INF-γ responsesDay 7: T-SPOT.®TB 2/16 (12.5%) negative to positive, 6/8 (75%) positive ↑ in INF-γ responses	IGRA negative subjects who boosted were TST positive.	Yes
Baker et al [27] 2009	USAimmigrants/ refugeesin US less than 6mo (mainly high burden countries)	114	5TU PPD-S	QFT-GIT	0, 14–112	<35 days: 2^nd^ IGRA 87%↑ in INF-γ responses,35 -112 days: 69%2^nd^ IGRA ↑ in INF-γ responsesIGRA positive 86% showed boosting*IGRA negative 68% showed boosting	*TST positive boosted by 82% whereas TST negative by 62% (p = 0.06)	Yes
Belknap et al [17] 2009 [abstract] #	USAHCWs (equal number of TST +/TST -) (Low)	125	5TU Tubersol	QFT-GITT-SPOT.TB	7–21^+^	QFT-GIT: 12 (10%) negative to positive,T-SPOT.TB: 12 (10%) negative to positive	Exact testing days not specified; Only IGRA negative recruited TST status did not predict boosting	Yes
Vilaplana et al. [23] 2008	SpainTB researchers (low)	9	2TU RT23	ELISPOT & WBA IFN-γ*	0,7, 14, 28	IGRA neg/TST neg 5–60 x ↑at day 7* (4 subjects)IGRA pos/TST pos 20–400 x ↑ at day 7* (3 subjects)IGRA pos/TST neg 5–80 x ↑ at day7 * (2 subjects)	* Depending on Antigen used; ^+^ Cellestis Ltd.	Yes
Choi et al [26] 2008	South KoreaHCWs in Pulmonary Medicine working >1 year (medium)	59	2TU RT23	QFT G	0, 14–28	Median IFN-γ responses ↑ at visit post TST0.05 to 0.19IU/ml increase in TST positive group (p = 0.01)IGRA neg/ TST pos 3/18 (16.7%) become IGRA positive IGRA neg/TST neg zero became positive (p = 0.11)		Yes
Perry et al [22] 2008	infectious disease cohort (low)	63	5TU Tubersol	QFT-GIT	0, 84 (3 mo)	Day 84: 3/48 (6%) QFT negative became positiveDay 84: Mean IFN-γ responses ↑ in initially QFT positive subjects	Non significant trend for inconsistent QFT results to be discordant by TST at baseline	yes
Richeldi et al [20] 2008 *	Italy Paediatric TB contacts	70 & 81	5TU PPD S	QFT-G/QFT-GIT	0, 56–77	QFT-G: 1/51(2%) negative became positive (no change in mean QFT levels in negative subjects)QFT-GIT 1/63 (1.5%) negative became positive		No
Leyten et al [18] 2007	The Netherlands Known TST 0mm (n = 15) and known TST ≥10mm (n = 51) (low)	66	2TU RT23	QFT GIT	0, 3,(10,11)*	Day 3: no categorical changesDay10: 1 negative to positiveDay11: 1 positive ↑ in INF-γ response	*Boosting shown only in two with delayed processing, and this was not statistically significant	No @ 3daysYes @ 10 days
Igari et al [25] 2007	JapanUniversity Medical students, Negative baseline QFT and TST <15 mm (low)	33	3TU PPD	QFT-G	0, 42	Day 42: IGRA neg/TST neg; 5(15%) became positive	Only concordant baseline negatives had second IGRA	Yes
Naseer et al. [24] 2007	UKSubjects not specified, No Hx of TB contact or disease (low)	10	Not reported	QFT-GT-SPOT.TB	0, 2, 42	Day 42: 3/9 (33%) QFT negative became positiveDay 42: 0 T-SPOT negative became positive	No qualitative results reported; No boosting if blood drawn at TST administration	Yes
Cellestis Ltd, Australia - QFT USA Package insert [28] 2007	USA	530	Not reported	QFT-GIT	0, 28–35	IGRA negative 3 became positive (total number of negatives not reported), 5 initially positive reverted	Industry study not published	No
Richeldi et al [19] 2006 *	Italy TB contacts (low)	44	5TU PPD S	T-SPOT.TB	0, 9, 15 24 months (Post TB exposure)	Month 24: all subjects remained IGRA negative, although 3 converted by TST	All subject TST and IGRA negative at first visit	No
Nguyen et al [21] 2005	USAinfectious disease cohort (low)	48	5TU Tubersol	QFT- TB	0, 84 (3 mo)	Day 84: 1/27 (4%) negative became positive (p = 0.10)	This study primarily investigated TST-TST boosting (PPD) responses) and discordance.	No

#updated preliminary data presented at the Second Global Symposium on IGRAs. Daley C. Evaluation of interferon-g release assays in the diagnosis of latent TB infection in US healthcare workers: preliminary results of Task Order #18. 31 May 2009; Second Global Symposium on IGRA, Dubrovnik, Croatia, 2009.

* Retrospective studies.

Four studies used 2TU RT 23 PPD, three used 5TU PPD-S, three used 5 TU tubersol, one used 3TU PPD (in two studies PPD type was not reported). Five studies used the T-SPOT.TB assay, 6 studies the QuantiFERON-TB Gold assay (various generations) and 4 studies had data using both IGRA platforms. The time points for assessing impact of TST varied widely. The range of time points used was from 3 days post-TST to 2 years after TST. Of the 13 studies, 5 concluded that boosting did not occur. [18], [19], [20], [21], [28] In four of these studies [19], [20], [21], [28] the earliest time point of repeat IGRA testing ranged from 28 days to 9 months. The other study by Leyten et al [18] used only day three results after TST and found no evidence of IGRA boosting. It is relevant to note that in this latter study two subjects inadvertently had the second IGRA on day 10 and 11 (instead of day 3) – both these subjects demonstrated boosting in responses.

Of the 7 studies that concluded that boosting does occur, 5 had repeat IGRA testing within 21 days after TST. Thus, it appears that the time point at which the second IGRA is done is highly relevant to the assessment of whether boosting occurs after TST. The TST used in the studies did not appear to correlate with boosting as boosting was documented in at least one study for each of the PPD reagents used.

Most of the studies included both IGRA-negative and positive subjects (at baseline) with variable TST status. However, two studies only recruited IGRA-negative subjects [17], [25] to undergo a second TST. IGRA-negative subjects in most studies (using the shorter time points) generally did not boost with only a small percentage boosting (2–12%). It is only possible from two of the studies to relate this to TST Status. In the study by van Zyl-Smit et al. [2] the IGRA negative subjects who boosted were all TST-positive. The study by Belknap et al. [17] concluded that TST status did not predict boosting.

Two studies reported on the quantitative IFN-γ levels pre and post TST. Perry et al. demonstrated a rise in mean IFN-γ levels in IGRA positive subjects post TST at day 84. This was reproduced by van Zyl-Smit et al. who showed a persistently elevated IFN-γ response up to day 84 for the cohort as a whole although some individuals had returned to pre- TST levels by day 28.

## Discussion

While IGRAs have emerged as promising alternatives to the TST, there is still controversy regarding the most effective strategy for their use. For example, some national guidelines recommend replacement of the TST with the IGRA. Some recommend that either TST or IGRA can be used (but not both), while several countries (e.g. Canada, UK, Italy, Germany, Switzerland, Netherlands, Korea and Norway) recommend a two-step approach of TST first, followed by an IGRA. In fact, a recent survey of global IGRA guidelines showed that the two-step approach appears to be the most favoured guideline recommendation worldwide. [30] Boosting, clearly, is a key concern with the two-step approach, and thus far, only the Canadian guideline has explicitly addressed this issue and recommended that blood be drawn for IGRA on or before the day when the TST is read [8].

The use of IGRAs for serial testing is also contentious, given the lack of clarity on how to interpret values close to the assay cut point and how to define and treat IGRA conversions and reversions. A “grey zone” exists for T-SPOT.TB values close to the cut point whereas the QFT-GIT does not and in addition, some countries recommend IGRAs for serial testing while others do not. Several studies from both high and low TB burden countries [31], [32], [33], [34], [35], [36], [37] now suggest that IGRA conversions and reversions occur frequently and there is no clear consensus on how to interpret and deal with such results. In this context, our systematic review provides useful insights into some of these issues.

### Within-Person Variability

There is a striking lack of published, peer-reviewed reproducibility studies that met our inclusion criteria, which is surprising, given that commercial IGRAs have been available for over 5 years now. Although some studies reported evaluating IGRA reproducibility, they were performed following tuberculin skin testing or in the context of contact screening and thus cannot be considered to be reproducibility studies. There were 3 published variability studies that investigated within-subject variability, i.e. serially testing the same individual over several days to weeks [2], [3], [16]. A fourth study by Belknap et al. [15] is currently ongoing (this study however only uses two time points.).

The three published reproducibility studies reported total only 67 subjects – although the total number of IGRA tests performed exceeds 350. It is difficult to compare these three studies - although they were all performed in high burden settings, the time points used were not the same. The study by van Zyl-Smit et al. [2] included assessment of both QFT-GIT and T-SPOT.TB assay – not previously reported.

**Figure 2:**
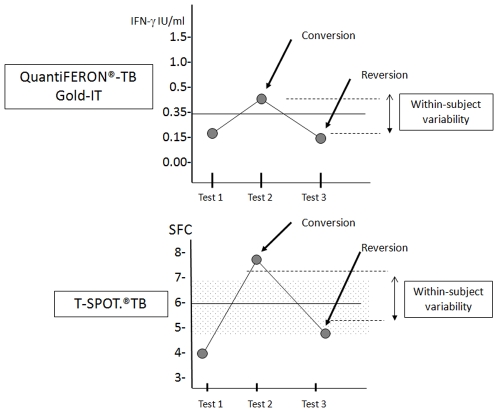
Schematic of the concept of “conversion and reversion” and “within-subject variability”. The conversion and reversion points depicted are based on the manufacture's definitions with a hypothetical within-subject variability or borderline/grey zone indicated. The shaded area for the T-SPOT.TB diagram is the FDA defined grey zone.

Regardless of the small samples and variability in methods and tests, these studies show that variability in IGRA results does occur and is not inconsequential in high burden settings. Variability is most frequently seen with baseline positive IGRA results, and in those results that are around the cut-off points. Anecdotally and in published reports, it is not uncommon to serially test individuals, especially those with values around the cut-off, and find their IGRA values cross the assay cut-point. Within-subject variability may explain most of these observations. Figure 2 is a schematic that attempts to capture this notion. From the available data, it is not easy to tease out the biological/host factors that result in within-subject variations, from laboratory and technical factors that can result in variations. Further work is needed to resolve these sources of variation. There are no published data regarding within-subject variability in low burden settings, but preliminary findings from an ongoing study in the USA [15] confirms the findings seen in high burden settings. Additional studies are needed in low TB incidence countries.

Given the variability seen in results from individuals undergoing repeat testing a “borderline”/grey zone for a single test value close to the cut-point appears reasonable for the T-SPOT.TB assay and was required for US Food and Drug Administration (FDA) licensure of T-SPOT.TB. It remains to be seen if the FDA defined grey zone or those newly proposed by independent researchers are clinically useful. For the QFT-GIT, although some variability has been shown, more data are required to accurately define the grey zone around the cut-point. It is not possible to propose a definitive grey zone for use by clinicians in all settings based on the available data. Large studies from high and low burden countries are needed to enable a meaningful estimation of the magnitude of variability in all settings.

### Boosting Effect of TST on IGRA Results

There are now a considerable number (12) of studies that have investigated the effect of the TST on subsequent IGRA results including an additional study undertaken by the US Navy and CDC, reported in the package insert for the manufacturer of the QFT assay (Cellestis Limited, Victoria, Australia). These studies however have used different generations of the various IGRA assays as well as using vastly different time points, range 3 days to 730 days, upon which to base their conclusions. These differences precluded any numeric pooling (meta-analysis). The conclusions about whether boosting of IGRA responses occurs after the TST also needs to be related to the initial IGRA or TST status of the individual.

In general, there is growing evidence that the TST can indeed boost subsequent IGRA results. However, the effect appears to be more apparent in those individuals who were already IGRA-positive to begin with (i.e. previously sensitized to *M. tuberculosis* or possibly other mycobacteria). Also, the effect seems apparent only after the first few days (day 3 post TST) and potentially wanes after 3 months, but this requires further confirmation. There are no data which allow us to predict when the boosting effect of TST is likely to wane.

Although the boosting studies presented in this systematic review could be considered to present contradictory evidence, this is probably not the case. All the studies that demonstrated boosting used time points between 7 and 28 days for the second IGRA (post TST.) The studies that showed no evidence of boosting generally had time points less than 7 days or greater than 3 months for the second IGRA. The crucial time point is clearly day three (time of TST reading) but future boosting studies must use multiple time points. To determine the “onset” of boosting studies would specifically need to examine days 1,2,3,4,5 and 6 and then multiple days beyond the first week, to ascertain how long the boosting effect might last occur.

The second important issue is to separate baseline IGRA-negative and IGRA-positive subjects. IGRA-positive subject show clear boosting in three studies. [2], [22], [23] This is biologically intuitive and perhaps expected as IGRA positive individuals likely have circulating memory T cells that have previously been exposed to RD-1 antigens. [2] This will in most clinical settings probably be irrelevant because IGRA-positive subjects are not likely to be re-tested in routine programs (just as TST-positive individuals are usually not re-tested with TST). However, in the context of following IGRA trends in response to TB treatment (e.g. as a biomarker for treatment response) or attempting to predict the risk of developing active disease, a TST may affect our ability to interpret serial IGRA test results.

In IGRA-negative subjects, the issue of boosting is most relevant and contentious. The major implications of whether boosting occurs or not, is to the two step strategy for IGRA testing of risk groups such as immigrants and household contacts. It is clear from the studies presented that only a smaller but not insignificant percentage of IGRA-negative individuals (2-12%) boost following a TST. However, the proportion may be larger as the published studies only enrolled small numbers of IGRA-negative subjects (range 12–51). The implication for this group is that they would receive inappropriate INH chemoprophylaxis on the basis of a falsely positive IGRA. It is further not clear, however, if only IGRA negative subjects whose TST is positive, boost with a resultant positive post-TST IGRA. Larger studies are required.

There are no published data documenting the exact amounts of RD-1 antigens/peptides contained in PPD formulations that are on the market. It is also not clear if the magnitude of the boosting effect is generalisable to all PPD formulations, although boosting has been documented for most commercial TST formulations.

There are insufficient data to determine if, and at what interval, boosted IGRA levels will predictably return to baseline after a TST. Current data suggests that if blood for IGRA testing is drawn before or within 72 hours of the TST being planted this should not result in false positive IGRA results due to boosting. Thus, it does appear that the optimal time to collect blood for IGRA is at the time of reading the TST. This approach has already been recommended in the Canadian guidelines[8]; other guidelines may need to be updated accordingly.

### Future Research Directions

It is clear that we need more data on reproducibility of IGRAs, both short-term as well as long-term. In particular, reproducibility studies of the two commercial assays are urgently needed, because they are most likely to be used in routine clinical practice. Studies in both high and low incidence settings are required as the results may differ due to the potential confounding of concurrent TB exposure. Better definition of a borderline/grey zone for the assay cut point will provide clinicians with more confidence when dealing with individuals who have values close to the cut-point. Existing package insert data and data used for FDA and other regulatory approvals do provide some reproducibility data, but they do not quite provide the longitudinal within-subject variability results that are needed for serial testing interpretation. In any case, independent studies are necessary for policy making, beyond the industry generated data.

Large prospective studies in both high and low burden countries are required to come up with definitive recommendations regarding the timing of TST and IGRA, and exact definitions for conversions and reversions. Such studies are ongoing. It will be important that these studies use a variety of commercially available PPD preparations and multiple time points prior to and following the TST. Until definitive recommendations can be made, it may be prudent to assume that IGRAs are dynamic tests that can produce variable results. So, borderline IGRA results should always be carefully interpreted with consideration of relevant clinical information. It is also prudent to assume that boosting of IGRA by TST is likely after the initial few days, although we still do not know how long such boosting effects last.
